# Editorial: Unfolded protein response (UPR): An impending target for multiple neurological disorders

**DOI:** 10.3389/fnagi.2022.1014450

**Published:** 2022-09-23

**Authors:** Arif Tasleem Jan, Safikur Rahman, Khurshid Ahmad, Rinki Minakshi

**Affiliations:** ^1^Gene Expression Lab, School of Biosciences and Biotechnology, Baba Ghulam Shah Badshah University, Rajouri, India; ^2^Department of Botany, Munshi Singh College, BR Ambedkar Bihar University, Muzaffarpur, Bihar, India; ^3^Department of Medical Biotechnology, Yeungnam University, Gyeongsan, South Korea; ^4^Department of Microbiology, Swami Shraddhanand College, University of Delhi, New Delhi, India

**Keywords:** aggregation, endoplasmic reticulum stress, neurological disorders, protein folding, proteinopathies, unfolded protein response

## Introduction

The endoplasmic reticulum (ER) is responsible for carrying out the process of folding, maturation, and trafficking of proteins, following their synthesis in the cell. The function of the ER can be disrupted by environmental insults, resulting in the accumulation of misfolded or unfolded cargo in its lumen, a condition referred to as ER stress. The ER normally has a rescue pathway to take care of the damaged or misfolded protein overload, which involves halting the translational machinery, upregulating the expression of the chaperones, and diverting misfolded or unfolded proteins to degradation pathways, called ER-associated degradation (ERAD). If this rescue pathway fails, the protein aggregates will be sensed by PERK1, IRE1, and ATF6 in the ER, resulting in the activation of an extremely conserved signaling pathway called the ER unfolded protein response (UPR^ER^). If the accumulation of misfolded proteins and the stress and damaging conditions associated with them persist for a long time, the resulting cellular injury can manifest itself in the form of a variety of etiologies in the brain, including Alzheimer's disease (AD), Parkinson's disease (PD), and amyotrophic lateral sclerosis (ALS). In this context, it has become increasingly important to: (a) decipher the pathophysiology of neurodegenerative disorders (NDs), (b) put up a multidimensional assessment of the genetic mechanisms underlying these disorders, (c) identify mechanisms that link stress responses to neuronal function, (d) set up preventive interventions to reduce the occurrence of these diseases, and (e) strategize pharmacological interventions to ameliorate the effects of neurological disorders, as an optimal method for extending the survival of affected individuals.

With our call for articles, we provided an interface for authors to share their views and further explore the fundamental aspects of the UPR^ER^ in the pathophysiological regulation of neuronal disorders. This Research Topic is a multifaceted collection of analyses contributed by clinicians, neuroscientists, and academicians. It highlights recent advancements in the methodological approaches, pharmacological interventions, and conceptual issues that can be of immense use in dealing with different neurological disorders. Our goal was to make valuable contributions to deciphering different aspects of the occurrence of neurological disorders. This includes interrogating the interplay of UPR^ER^-based signaling modules and the mechanism of their entanglement with neuronal abnormalities, as well as offering perspectives on designing different therapeutics against neurological diseases.

This issue contains review (five articles) and original research (four articles) covering recent advances on different aspects of the ER unfolded protein response and protein aggregation, and a series of papers covering different aspects of the physiological and pathological consequences of UPR^ER^ that lead to the development of NDs.

Zhao et al. review the complex interplay of ER stress and UPR^ER^ in ALS and TDP-43-associated pathology. ALS, a disease characterized by degeneration of the motor system, was found to exert its effects through dysregulation of the proteostasis, progressing through the aberrant build-up of misfolded/unfolded proteins in the ER lumen.

The article by Yasmeen et al. focuses on deciphering the link between UPR^ER^ signaling and microRNA in the pathogenesis of AD. In their study, they extensively elaborated ER stress and UPR in the dysregulation of microRNAs and the resulting consequences in the pathogenesis of AD.

The article by Alam and Akhter studies the occurrence diabetic retinopathy, a challenging eye disease, and then discusses its association with ER stress and UPR^ER^. The authors also point out the key cellular programs in the development of diabetic retinopathy, thus providing a better understanding and elaborative view of the molecular and cellular basis of the development of this condition.

Fernandez et al. sought to explore this association further by investigating the role of UPR^ER^ in the development and function of immune cells. In this article, the authors studied the capacity of UPR^ER^ to set the threshold between cell survival and death and tried to establish the potential contributions of UPR^ER^ in brain-associated immune cells in the context of the development of an ND. This topic is discussed further in the article by Mukherjee et al. who reviewed the literature and summarized observations to ultimately present evidence that curcumin slows the progression of ND. The study established a relationship between curcumin levels, UPR^ER^, and chance of ND development.

The original articles submitted to this issue present a range of relatively simple and pragmatically implementable pharmacological approaches toward promoting health and improving interventional strategies as part of reducing the development of ND. Pathak et al. studied the role of ofloxacin (a broad-spectrum antibiotic) as a possible drug molecule for use in the treatment of ND. The study both employed *in silico* modules and made use of biophysical techniques to uncover the dynamics of actin polymerization. The authors report that actin polymerization is disrupted upon binding with ofloxacin in a concentration-dependent manner, and thus holds great promise for use in the treatment of neurological disorders.

Warepam et al. address differences in the deposition levels of toxic protein inclusions in the presence and/or absence of an important brain metabolite, N-acetylaspartate (NAA). They report that NAA, whose levels have been reported to be altered in a variety of neurological disorders, acts as a potential protein stabilizer with the capacity to inhibit the aggregation of carbonic anhydrase and catalase. However, the authors debate whether an increase in alpha-synuclein is fueled by decreasing concentrations of NAA. They propose that NAA, with its potential to suppress protein aggregation, may offer benefits in terms of solubilizing the protein aggregates associated with the development of NDs.

Chen et al. integrated experimental and computational methods to show the expression pattern and potential implications of ER stress in the development of intracranial aneurysm (IA). The authors observed that the majority of the differentially expressed genes in the Gene Expression Omnibus and RNA sequencing datasets they studied are associated with ER stress, autophagy, and metabolism. The authors predicted a total of nine targets, all associated with ER stress, that can act as potential drug targets for use in delaying the formation and progression of IA.

Li et al. examined the neuroprotective effects of exercise after stroke and elucidated the role of SIRT1 in attributing neuroprotection. The authors used an ischemia/reperfusion rat model to investigate the effects of a post-stroke exercise regimen and its potential to regulate reactive oxygen species (ROS)/ER stress through the involvement of SIRT1. They established that SIRT1 is associated with the regulation of neuronal functioning and brain health and elucidated that mild exercise post-stroke, as well as intense exercise, might play a beneficial role in attributing neuroprotection. This comes as a significant finding in that the inclusion of exercise and regular follow-up assessments can be understood as a preventive step against the development of ND.

## Conclusion

The articles published under this Research Topic cover a broad range of subjects that are directly related to the pathogenesis of ND, as well as its diagnosis and potential therapeutics. Other articles highlight the role and functionality of UPR^ER^ as well as lifestyle changes that affect the development of ND.

## Author contributions

AJ prepared the initial draft of this editorial. SR, KA, and RM carefully revised the draft. All authors contributed to the contents of this article and approved the final version.

## Funding

This work was supported by the Department of Science and Technology, India under Science and Engineering Research Board (DST-SERB) Grant No. CRG/2019/004106 and J&K Science Technology and Innovation Council (J&K ST&IC), India Grant No. (JK ST&IC/SRE/996-998).

## Conflict of interest

The authors declare that the research was conducted in the absence of any commercial or financial relationships that could be construed as a potential conflict of interest.

## Publisher's note

All claims expressed in this article are solely those of the authors and do not necessarily represent those of their affiliated organizations, or those of the publisher, the editors and the reviewers. Any product that may be evaluated in this article, or claim that may be made by its manufacturer, is not guaranteed or endorsed by the publisher.

